# Ethanolic Extract of *Salvia hispanica* L. Regulates Blood Pressure by Modulating the Expression of Genes Involved in BP-Regulatory Pathways

**DOI:** 10.3390/molecules25173875

**Published:** 2020-08-26

**Authors:** Gerardo I. Arredondo-Mendoza, Zacarías Jiménez-Salas, Francisco Javier Guzmán-de la Garza, Elizabeth Solís-Pérez, Manuel López-Cabanillas-Lomelí, Blanca Edelia González-Martínez, Eduardo Campos-Góngora

**Affiliations:** 1 Universidad Autónoma de Nuevo León, Centro de Investigación en Nutrición y Salud Pública, Eduardo Aguirre Pequeño/Yuriria Str. Monterrey 64460, Nuevo León, Mexico; gerardo.arredondom@uanl.mx (G.I.A.-M.); zacarias.jimenezs@uanl.mx (Z.J.-S.); elizabeth.solis@uanl.mx (E.S.-P.); manuel.lopezc@uanl.mx (M.L.-C.-L.); 2 Universidad Autónoma de Nuevo León, Facultad de Medicina, Av. Madero, Monterrey 64460, Nuevo León, Mexico; fcojguzman@hotmail.com

**Keywords:** *Salvia hispanica* L. extract, bioactive compounds, hypertension, gene expression, rats

## Abstract

Hypertension (HT) is considered to be a potential risk factor for cardiovascular diseases and has been directly related to pathologies such as obesity and dyslipidemias. Angiotensin-converting enzyme inhibitors (ACEIs) blocked the renin-angiotensin-aldosterone cascade diminishing the production of angiotensin II and the level of bradykinin, produced by the kallikrein-kinin system. Although ACEIs are effective therapeutics in regulating HT, they present several side-effects that can be due to their mechanism of action (as hypotension, cough, dizziness, light-headedness or hyperkalemia) to specific drug molecular structure (skin rash, neutropenia and tasting disorders) or due to associated pathologies in the patients (it has been considered a possible nephrotoxic effect when ACEIs are administered in combination with angiotensin receptor blockers, in patients that present comorbidities as diabetes, acute kidney injury or chronic kidney disease). Therefore, it is necessary the searching for new products with ACEI activity that do not produce side effects. Interestingly, species of the plant genus *Salvia* have been found to possess hypotensive effects. In the present study, we analyzed the effects of the ethanolic extract of *Salvia hispanica* L. seeds (EESH) on the expression of genes involved in pathways regulating HT. Administration of EESH to hypertensive rats inhibited the angiotensin-converting enzyme (ACE) activity along with a decrease in *Ace* and elevation of *Agtr1a* and *Nos3* gene expression, as compared to that in healthy rats. Moreover, these results were similar to those observed with captopril, an antihypertensive drug used as a control. No significant change in the expression of *Bdkrb2* gene was observed in the different groups of rats. To conclude, our results demonstrate that EESH regulates blood pressure (BP) in hypertensive rats through transcriptionally regulating the expression of genes that participate in different pathways involving ACE.

## 1. Introduction

Hypertension (HT) or high blood pressure (HBP) is one of the most common conditions observed in primary care setting worldwide and may lead to myocardial infarction, stroke, renal failure and death, if not detected early and treated appropriately [1]. Arterial pressure has been regarded as the key factor in regulating the cardiovascular system, with important health implications [2]. For example, abnormal changes in systolic and diastolic blood pressures (BPs) act as long-term risks for developing chronic heart disease and cardiovascular disease, finally resulting in a progressive and linear increase in the risk of death from heart disease and stroke [3]. The renin–angiotensin system is an important hormone system that regulates arterial BP and maintains the electrolyte homeostasis. This pathway involves conversion of angiotensinogen by renin into its biologically inactive decapeptide angiotensin I (Ang-I), which is then cleaved by the angiotensin converting enzyme (ACE) to Ang-II, the primary active peptide of the system [4]. Ang-II functions by sequentially removing two C-terminal dipeptides and inactivating the vasodepressor bradykinin [5]. Another biological mechanism regulating BP is the synthesis of nitric oxide (NO), which is recognized as a pivotal endogenous modulator of vascular tone and endothelial function [6].

Angiotensin converting enzyme inhibitors (ACEIs) and angiotensin receptor blockers (ARBs) are widely prescribed for treating primary HT. However, while ACEIs have been shown to reduce mortality and morbidity in placebo-controlled trials, ARBs have not. Therefore, a comparison of the efficacies of these two drug classes in primary HT for preventing total mortality and cardiovascular events is important [7]. Numerous bioactive compounds have been reported to possess hypotensive effects. For example, phenolic compounds with anti-ACE activity are seen as promising alternatives to synthetic drugs [8].

*Salvia* is a genus of about 900 species of plants of the Lamiaceae family. Chia (*Salvia hispanica* L.) is a representative of this genus that has been used since ancient times for dietary and medical purposes [9]. This species grows from Western Mexico to Northern Guatemala, although recently it has been cultivated in other countries as Argentina, Chile, New Zealand, Japan, USA, Canada and Australia, due principally to the European Parliament declaring chia as a functional food [10,11].

Various bioactive compounds have been identified in the *Salvia hispanica* L seeds. In the recent years, there have been many discussions and studies about the health benefits and use of chia seeds, which are a rich source of nutrients such as carbohydrates, dietary fiber, proteins, vitamins and minerals. In addition, to possessing a high content of compounds that provide health benefits beyond basic nutrition, such as antioxidants, and polyunsaturated omega-3 fatty acids [9]. Additionally, the presence of these potential bioactive compounds in *S. hispanica* seeds confers therapeutics properties such as anti-inflammatory effects [12], antimicrobial and antiproliferative activities [13], enhance cognitive performance, reduce the level of cholesterol and regulates bowel function [14]. As reported in several clinical studies, chia consumption has been related to antihypertensive effects, prevention of overweight/obesity, increase of plasma adiponectin, decrease of biochemical variables of metabolic syndrome, improvement in lipid profile and hyperglycemia values, all of them are associated parameters to cardiovascular disease. Although scientific evidence appoint that several of the observed effects are due to the antioxidant activity of compounds presents in *S. hispanica* seeds, it has been mentioned that further studies need to explore more about the mechanism of biochemical activities associated with chia using mechanistic approaches in cell and mammal models (for important reviews see [9,10,11,14,15]).

With regard to antihypertensive effects, chia (*Salvia hispanica* L.) seeds contain compounds, such as myricetin, quercetin, kaempferol and caffeic acid, with potent antioxidant activity [16,17,18]. Besides, its seed extracts have been demonstrated to contain chemical compounds such as glycosides of terpenes and glycosides linked to phenolic compounds, which exert significant ACE inhibitory and hypotensive activities, as evident from in vitro and in vivo studies, respectively [18,19,20]. Similarly, administration of extracts of Danshen (the dried roots of *Salvia miltiorrhiza* Bunge) to a human endothelial umbilical cell line increased the expression of the gene coding for the endothelial nitric oxide synthase (eNOS), the activity of the eNOS promoter and the production of endothelial NO. The presence of ursolic acid, a pentacyclic triterpene hydrophilic and/or soluble in alcohol and commonly present in Salvia plants, was found to be the active constituent responsible for these effects [21]. In addition, other studies have shown that Salvia extracts rich in phenolic compounds are effective in controlling HT through a mechanism involving inhibition of ACE. In the present study, we analyzed the effects of an ethanolic extract of *S. hispanica* L. seed (EESH) on the expression of genes involved in the HT-regulatory rennin–angiotensin pathway in a hypertensive rat model.

## 2. Results

### 2.1. Inhibitory Activity of Angiotensin Converting Enzyme In Vitro

The in vitro assays revealed the ACE-inhibitory activity of EESH to have a linear relation with the amount of the extract used; the maximal inhibitory activity on ACE was obtained using 200 mg/mL of EESH (58.4%), whereas the maximal ACEI reached with captopril (100 mM) was 66.2%. In general, all tested EESH concentrations (mg/mL) showed ACE inhibitory effect: 25 (35.6%), 50 (42.1%), 75 (47.2%), 100 (49.2%), 150 (51.9%) and 200 (58.4%). On the other hand, captopril (100 nM) resulted in 66.2% inhibition of ACE activity. With the values obtained from the dose–response curves, the concentrations required to inhibit 50% of ACE activity (IC50) were calculated, both for the EESH (9.37 mg/mL) and captopril (0.0142 µg/mL). For the in vivo assays, the EESH stock solution (200 mg/mL), which showed a higher ACEI effect, was used.

### 2.2. Induction of Hypertension and Effect of Antihypertensive Treatments

Administration of L-NAME resulted in an increase in BP values in rats. BP in the hypertensive rats reached values above 300 mmHg (mean, 318.66 mmHg), whereas BP values in the control group were maintained at 100 ± 5.4 mmHg. After antihypertensive treatments, BP values in hypertensive rats (rats without antihypertensive treatment, but with continuous L-NAME administration) demonstrated an increase of 31.2%, reaching above 400 mmHg (414.1 ± 7.1 mmHg). On the other hand, although rats receiving antihypertensive treatments (captopril or EESH administration plus continuous L-NAME administration) did not show a significant decrease in BP, the BP values were maintained at 327.8 ± 8.7 and 317.7 ± 8.1 mmHg in rats treated with captopril and EESH, respectively (Table 1).

### 2.3. Effect of Antihypertensive Treatments on Gene Expression

Expression patterns of genes encoding proteins implicated in the regulation of BP were determined by RT-PCR. The primers used are listed in Table 2. The expression level of each gene was normalized relative to that of the reference gene *Gapdh*. Changes in the levels of expression of genes in different groups were calculated with respect to the expression levels of these genes in the healthy rats.

#### 2.3.1. *Ace* Gene Expression

**Figure 1:**
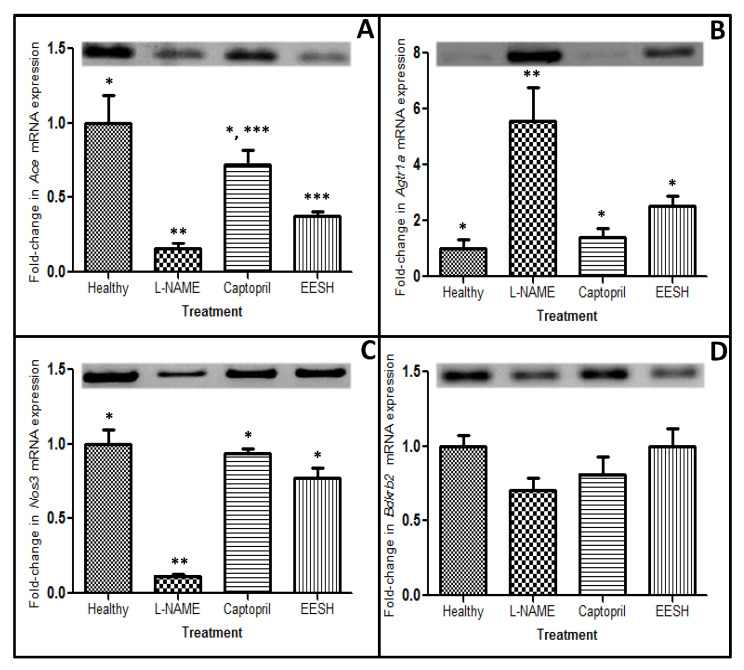
Comparison of the expression of *Ace* (**A**), *Agtr1a* (**B**), *Nos3* (**C**) and *Bdkrb2* (**D**) genes in hypertensive rats subjected to different treatments. Data are normalized to the *Gapdh* gene and fold changes between treatments are expressed as mean ± standard deviation. Upper panels show representative gel bands. Different symbols (*, ** or ***) represent significant differences (ANOVA test; *p* ≤ 0.05). ANOVA: analysis of variance, EESH: ethanolic extract of *Salvia hispanica* L. seeds; L-NAME: N-nitro-L-arginine methyl ester.

The *Ace* gene expression was lower in the hypertensive rats (L-NAME group), as compared to its expression in the healthy rats, showing a decrease until 6.4-folds (*p* < 0.001). Statistical analysis showed that values of *Ace* gene expression in the group treated with captopril were equal to that in the group of healthy rats (*p* = 0.750). Interestingly, levels of *Ace* gene expression were equal (*p* = 0.076) between groups treated with either captopril or EESH, and different (*p* < 0.001) when compared to the hypertensive rats. With respect to the last group, *Ace* gene expression increased 4.6-fold and 2.4-fold after the treatment both with captopril or EESH, respectively (Figure 1A).

#### 2.3.2. *Agtr1a* Gene Expression

Contrary to the expression level of the Ace gene, the expression of the *Agtr1a* gene was higher in hypertensive rats, showing an increment of 5.56-folds (*p* = 0.002), with respect to the healthy group. When captopril or EESH was administered, the *Agtr1a* gene expression decreased significantly (4.0-folds and 2.2-folds, respectively; *p* < 0.05); reaching values similar to the control group (*p* = 0.93 and *p* = 0.43, respectively; Figure 1B).

#### 2.3.3. *Nos3* Gene Expression

Hypertensive rats showed a decrease in the expression of *Nos3* gene until 8.4-folds (*p* = 0.007) as compared to the control group (healthy rats). After administration of captopril or EESH to the hypertensive rats, *Nos3* gene expression increased significantly (*p* < 0.001) by 7.8-folds and 6.4-folds, respectively. Statistical analysis revealed mRNA levels of *Nos3* to be equal (*p* = 0.891) to that of the control group in hypertensive rats subjected to any of the treatments (captopril or EESH; Figure 1C).

#### 2.3.4. *Bdkrb2* Gene Expression

We did not observe any significant difference in the expression of *Bdkrb2* gene in the treated groups, with respect to the control group (Figure 1D) although hypertensive rats demonstrated the lowest gene expression values (0.71-folds), followed by captopril and EESH treatment (0.81- and 0.99-folds, respectively).

## 3. Discussion

Angiotensin-converting enzyme inhibitors have been commonly used as a therapeutic to regulate HT. However, these drugs have been demonstrated to be associated with potential side-effects. To counteract this shortcoming, plant-based products have been increasingly tested for their antihypertensive effects. For example, plants belonging to the genus *Salvia* have been reported to inhibit ACE activity owing to the presence of a high content of aromatic compounds in it with antioxidant functions [22], such as caffeic acid, rosmarinic acid and lithospermic acid [23,24,25,26]. *S. hispanica* is a plant with interesting properties that can mimic synthetic ACEIs and provide potential health benefits, without side-effects. Besides, *S. hispanica* seeds have been used as an important dietary component by different communities, principally in countries of Latin America. A previous in vitro study by our group demonstrated EESH to exert a concentration-dependent inhibitory effect on ACE activity. Additionally, caffeic acid was found to be one of the main phenolic compounds present in EESH [18]. Interestingly, the ability of caffeic acid containing phenolic compounds to inhibit ACE activity has also been identified [26], which could explain the antihypertensive effect exerted by EESH. In the present study, we analyzed the effect of EESH on the expression of different genes implicated in the regulation of HT in a hypertensive rat model. We employed captopril, a pharmacological compound routinely used for treating HT, as the positive control for the inhibitory activity against ACE. Although the effect of captopril has been reported to vary depending on its method of synthesis and interaction of its excipients [27,28,29], captopril IC50 values in our experimental conditions were observed to be in the ranges reported in the literature for this compound (0.021–0.58 μM). The results obtained from the inhibitory effect of ACE, both with captopril and EESH, permitted us to define the adequate concentrations to be used in the in vivo assays, i.e., BP control and gene expression assays.

The in vivo assays revealed that administration of EESH inhibited the increase in BP in the hypertensive rats, an effect similar to that observed with the administration of captopril. Hypotensive effects have been observed upon treatment with isolated fractions of plant extracts, mainly associated with the presence of compound antioxidants, saponins and peptides [17,24,30]. The mode of action of the inhibitory effect is proposed to be the interaction of antioxidant molecules with different motifs on the ACE catalytic site [28], specifically, the union of these compounds with the zinc atom present in the catalytic site of ACE, as described by Reeves and Rossow [31]. EESH has been known to contain a high content of antioxidant compounds (chlorogenic acid, caffeic acid, myricetin, quercetin and kaempferol) [18], implying that the inhibitory effect of Salvia extracts could be attributed to the presence of antioxidants compounds. The finding by our research group that *S. hispanica* L. seeds contain compounds such as myricetin, quercetin, kaempferol and caffeic acid, having potent antioxidant activity, corroborate this hypothesis. Additionally, seed extracts of *S. hispanica* L. were demonstrated by in vitro and in vivo studies, respectively, to contain chemical compounds such as glycosides of terpenes and glycosides linked to phenolic compounds, with both ACE inhibitory and hypotensive effects [18,20].

In a similar way, the search for Salvia-based compounds with alternative mechanisms of action has resulted in the observation of changes in the expression of genes involved in the regulation of BP. For example, Steinkamp-Fenske et al. [21] and Zhou et al. [32] analyzed the effect of administration of *S. milthiorriza* extracts on the regulation and production of NO in a human umbilical endothelium-derived cell line. The authors found a concentration-dependent response besides an over-regulation of eNOS in the treated cells, as compared to control cells [21,22,23,24,25,26,27,28,29,30,31,32]. We analyzed genes coding for proteins that participate in three different pathways involved in regulation of HT, namely renin–angiotensin system (*Ace* and *Agtr1a* genes), the kallikrein–kinin system (*Bdkrb2* gene) and the nitric oxide (*Nos3* gene) synthesis pathway.

Hypertensive rats treated with L-NAME showed an important reduction in the expression of *Ace* and *Nos3* genes as compared to healthy rats. Administration of EESH led to notable changes in the expression levels of these genes, similar to that observed with the administration of captopril. This effect is comparable to that observed by Kobayashi et al. [33] who described that increased expression of *Ace* and *Nos3* genes following administration of imidapril to rats with hypertension induced by L-NAME; authors described that it could be attributed to a change in different systems responsible for regulating BP, as a compensatory response to the synthesis of ACE and the uptake of Ang-II by the receivers. Blocking of ACE by inhibitors induces a decrease in Ang-II production, stimulating the production of NO and bradykinin (molecules with a marked vasodilatory effect). NO, in turn, positively modulates the vascular system [33].

Experiments using L-NAME have demonstrated it to modify several biological processes, for example the synthesis of Ang-II is relevant owing to its ability to stimulate the release of NO, which influences the production of ACE and the binding of Ang-II to its receptors (AT1 and/or AT2). In the study conducted by De Gennaro et al. [34], HT in rats induced with L-NAME for 8 weeks resulted in a significant decrease in expression of *Nos3* gene, consequently reducing the production of NO and disrupting the dynamics of regulation of BP. Although eNOS was initially characterized as a constitutively expressed enzyme, its expression has recently been shown to change in response to physiological stimuli [35]. Additionally, Wallerath et al. [36] reported that compounds with potential antioxidant activity, such as cinnamic acid, cyanidin, p-coumaric acid, caffeic acid and benzoic and vanillic acids could effectively increase the expression of eNOS.

Changes observed in the expression of *Ace* and *Nos3* genes after administration of EESH are suggestive of a mechanism of action similar to that of captopril. The observation that EESH administration exerts a similar effect, producing an increase in the expression of both genes to values equal to that found in rats treated with captopril strengthens this hypothesis. Although the increase in the gene expression values was not equal to the values observed in healthy rats, a higher dose of extract could possibly result in a greater expression of *Ace* and *Nos3* genes.

The hypertensive rats also showed an increase in the expression of the *Agtr1a* gene, as compared to healthy rats. This could be attributed to decreased production of Ang-II and AT2 receptors, which, in turn, decreases the synthesis of NO and ACE. A study also reports that a blockage in AT1 receptor synthesis inhibits the negative feedback circuit that causes AT1 receptor to inhibit the secretion of renin from the juxtaglomerular cells, leading to increased renin secretion and Ang-II levels [37]. These data also agree with the results obtained by Hara et al. [38], which reported that the expression of *Agtr1a* gene was increased in hypertensive rats. In our work, administration of captopril or EESH to hypertensive rats decreased the expression levels of *Agtr1a* gene until it reached values similar to those observed in healthy rats. Similar effects were observed in other studies upon administration of antihypertensive drugs and synthesis of angiotensin receptors. In the study by Hara et al., involving the use of quinapril (an ACE inhibitor) to treat HT-mediated renal damage, expression levels of the *Agtr1a* gene were found to be similar to those exhibited by healthy rats [38].

We did not observe a change in the expression of *Bdkrb2* gene in rats belonging to different treatment groups (healthy, hypertensive, captopril treated and EESH treated). Moreover, *Bdkrb2* gene encoding for the bradykinin type 2 receptor of the Kallikrein–Kinin system has been proposed as a housekeeping gene [38,39,40,41]. The results presented here coincide with this postulation.

The ability of ESSH to modulate the expression of *Ace*, *Nos3* and *Agtr1a* genes, along with its in vitro inhibitory activity against ACE, makes it (1) an essential dietary component and (2) a potent adjuvant in treating HT and cardiovascular diseases.

## 4. Materials and Methods

### 4.1. Origin and Characteristics of Chia (Salvia hispanica) Seeds

In the current study organic chia seeds from state Colima in the western region of Mexico, which are commercialized by Xiomega 3 (Guadalajara, Mexico), were used. The seed was of the pint variety. The chemical composition of seeds was assessed in previous research works in our laboratory [42]. In these analyses, we found that the content of different chemical components as dry matter (1.26%), protein (23.53%), fat (26.62%), ash (4.55%), crude fiber (21.75%) and carbohydrates (16.95%) were consistent with the specifications recently published by the European Food Safety Authority [43].

### 4.2. Preparation of Ethanolic Extracts of Salvia hispanica L. Seeds

Chia (*S. hispanica* L.) seeds (50 g) were ground, mixed and homogenized in 400 mL of hexane (HPLC grade, ≥95%, CTR, Monterrey, México). After 24 h of continuous stirring, the material was filtered through a paper filter (Whatman No. 1). The recovered solid material was again subjected to maceration under described conditions, replacing hexane with 250 mL of ethanol and extending the maceration time to 72 h. The macerate was filtered and concentrated using a rotary evaporator (Hahnvapor HS-2001, Kyonggi, Korea) at 40 °C and 100 rpm until a final volume of 10 mL was obtained. The extract (stock solution) was stored at −20 °C.

### 4.3. Quantification of Angiotensin Converting Enzyme Inhibitory Activity In Vitro

Effect of EESH on the enzymatic activity of ACE was determined using the method established by Wu et al. [44]. This method is based on the degradation of the compound Hipuril-L-Histidil-L-Leucine (HHL) by ACE followed by quantification of the product of this reaction (hippuric acid) using high performance liquid chromatography (HPLC). To quantify hippuric acid (HA), a calibration curve was prepared using a 98% solution of HA as a standard (Sigma-Aldrich, St. Louis, MO, USA), dissolved in borate buffer (100 mM borate, 150 mM NaCl, pH 8.1) to obtain a final concentration of 25–100 mM.

To analyze the inhibitory activity of EESH, 6 different concentrations of EESH (25, 50, 75, 100, 150 and 200 mg/mL, prepared by dissolving EESH stock solution (200 mg/mL) in borate buffer 100 mM) were used. Samples were prepared by mixing 10 µL of EESH solution, 10 µL of ACE, isolated from the rabbit lungs (40 mU; Sigma-Aldrich, St. Louis, MO, USA) and 40 µL of HHL solution (2 mM; Sigma-Aldrich, St. Louis, MO, USA); these were then incubated at 37 °C with shaking (300 rpm) for 60 min. The reaction was inactivated by adding 50 µL of HCl (2 N) and then filtered (Acrodisc; 45 μm, PALL, Port Washington, NY, USA). The HHL content was analyzed by HPLC using the conditions described by Eriz et al. [12] and modified as per the method of Ortega [11]. Briefly, 20 µL of each sample was analyzed in an HPLC system (Thermo Scientific, Spectra System, Waltham, MA, USA), equipped with an ultraviolet/visible (UV/Vis) detector (Thermo Scientific, Spectra System, Waltham, MA, USA). The samples were separated by chromatography using a Syncronis C-18 RP column (150 mm × 4.6 mm, 5 μm; Restek, Bellefonte, PA, USA) and an isocratic elution 75% solution A (0.05% trifluoroacetic acid in deionized water) and 25% solution B (0.05% trifluoroacetic acid in acetonitrile), with a flow rate of 0.70 mL/min. The absorbance of samples was determined at 228 nm. As a positive control for inhibitory ACE effect, a dose–response curve was prepared under same conditions using 5–100 nM captopril (pharmacological grade). All reagents and solutions used were either of HPLC or analytical grade. IC50 values were calculated using ED50plus v1.0 online software.

### 4.4. Hypertensive Animal Model

To induce HT, 12 8-week-old male Wistar rats, weighing 250 g, were treated with N-nitro-L-arginine methyl ester (L-NAME; 40 mg/kg/day) [45,46]. Four rats receiving distilled water comprised the control group (without HT). After 6 weeks of treatment, BP of rats was determined using a non-invasive blood pressure device (NIBP); pulse signals were digitized using the LabChart 7 software (ADInstruments, Dunedin, New Zealand). Rats were considered hypertensive if a systolic blood pressure (SBP) ˃ 300 mmHg was recorded. Groups consisting of four animals each were formed, and antihypertensive treatments were initiated. These treatments consisted of daily administration (during 4 weeks) of: group (A), captopril (50 mg/kg) [39]; group (B), EESH (200 mg/kg) and group (C), without antihypertensive treatment (considered as the group with HT). In all groups, daily administration of L-NAME (40 mg/kg) continued until the end of the study. The last group (group D: rats with normal BP) served as the control, without HT. The SBP was measured after 4 weeks of antihypertensive treatment. Rats were euthanized by the guillotine decapitation method, following the guidelines of the Guide to the Care and Use of Experimental Animals of the Canadian Council on Animal Care (CCAC 1998).

### 4.5. Identification of Sequences Encoding Ace, Agtr1a, Nos3, Bdkrb2 and Gapdh Genes

Gene sequences were obtained from databases of National Center of Information and Biotechnology (NCBI; https://www.ncbi.nlm.nih.gov/) and the Universal Protein Resource (UniProt; http://www.uniprot.org/). Exons and introns were identified using the ORF finder tool (https://www.ncbi.nlm.nih.gov/orffinder/) to select encoding regions. After identifying the sequences, specific oligonucleotides were designed for the analysis of gene expression by reverse transcriptase-polymerase chain reaction (RT-PCR).

### 4.6. Gene Expression Analysis

Transcriptional analysis was performed by semi quantitative RT-PCR. For mRNA extraction, cardiac tissue (200 mg) was cut into small pieces (2 mm diameter), and lysed using 0.2 mm glass beads in 400 µL of TNST buffer (10 mM Tris base at pH 8.0, 100 mM NaCl, 1% sodium dodecyl sulphate (SDS), 2% Triton X-100 and 1 mM EDTA at pH 8.0), supplemented with 1% β-mercaptoethanol. RNA was purified using the Isolate II RNA Mini Kit (Bioline, London, UK) as per the manufacturer’s instructions, followed by quantification using the NanoDrop 2000 spectrophotometer (Thermo Scientific, Spectra System, Waltham, MA, USA). RNA samples were treated with DNase (Invitrogen, Carlsbad, CA, USA), and reverse transcribed using the GoScript Reverse Transcriptase System (Promega, Madison, WI, USA). Polymerase chain reactions (PCRs) were performed by conventional methodology using the RT-products, DNA polymerase (MyTaq, Bioline, London, UK) and appropriate primers. PCR products were resolved by electrophoresis in 2% agarose gels, stained with ethidium bromide and imaged using the GelDoc-It Imaging System UV analyzer (UVP, Cambridge, UK). Densitometry was performed using the Launch VisionWorks LS software (GelDoc-It, UVP, Cambridge, UK). Specific gene expression was normalized to that of the rat *Gapdh* gene, and changes in the treatments were calculated with respect to that in healthy rats.

### 4.7. Statistical Analyses

For the descriptive analysis of variables, means and standard deviations (SDs) were calculated. Statistical analyses were performed using the statistical software package IBM SPSS Statistics V21.0. Differences between treatments were analyzed using the analysis of variance (ANOVA) test. The statistical significance was determined at *p* < 0.05. Data corresponding to each variable are expressed as means ± SD, unless otherwise indicated.

## 5. Conclusions

The hypertensive rat model generated by L-NAME has been widely employed to evaluate the effects of different antihypertensive molecules [45,46]. In the present study, we aimed to assess whether ethanolic extract obtained of chia seeds (EESH) has antihypertensive activities.

Our results demonstrated that EESH exerted an inhibitory effect on ACE in vitro, similar to that exerted by captopril on the regulation of BP in hypertensive rats. In addition, a regulatory effect on the expression of other genes involved in HT-regulatory pathways was observed in rats treated with EESH and also similar to the effect produced by the administration of captopril.

The higher effect of EESH was observed in the modulation of *Ace, Nos3* and *Agtr1a* genes. The products of translation of these genes have been implicated in BP-regulating mechanisms [47,48,49]. On the other hand, the expression of *Bdkrb2* gene did not show changes in their expression and similar results were obtained when captopril was used for the antihypertensive treatment. The results suggest that bioactive compounds present in the EESH are able to modulate the transcription of specific genes, implicated in BP-regulatory pathways; so, the EESH could serve as a potential candidate as an adjuvant for treating HT. Moreover, the inclusion of whole *S. hispanica* L. seeds in the diet will promote the potential consumption of functional ingredients, in agreement with the recently recommended, and approved, by Panel on Nutrition, Novel Foods and Food Allergens of the European Food Safety Authority [43]. Notwithstanding, in future research it will be necessary to determine whether compounds purified from these extracts or a mix of them have the same antihypertensive effect, or rather, the synergistic action of its components is required to produce the effect observed by the EESH. This last assumption would prove that the consumption of the whole chia seed has a greater impact on promoting health than the consumption of its isolated components could have.

## Figures and Tables

**Table 1:** Systolic blood pressure in Wistar rats.

Groups	Pre-Treatment SBP (mmHg)	Post-Treatment SBP (mmHg)
Healthy	100.0 ± 5.4 ^ab^	86.7 ± 9.6 ^be^
L-NAME	310.7 ± 27.3 ^cd^	414.1 ± 7.1 ^f^
Captopril	328.2 ± 26.2 ^cd^	327.8 ± 8.7 ^dh^
EESH	318.3 ± 22.1 ^cd^	317.7 ± 8.1 ^dh^

SBP: systolic blood pressure, L-NAME: N-nitro-L-arginine methyl ester, EESH: ethanolic extract of *Salvia hispanica* L. seeds, SD: standard deviation. Mean ± SD of three independent measurements of SBP for each group of rats before and after antihypertensive treatments are reported. Different superscripts indicate significant differences (*p* ≤ 0.05) between groups.

**Table 2:** Primer specifications and conditions used for semi-quantitative reverse transcriptase polymerase chain reaction.

Gene	NCBI-RS	Primer Sequence (5′→3′)	Annealing Temperature (°C)	Amplicon Size (bp)
*Ace*	NM_012544.1	Fw: CAGGAGTTTGCAGAGGTCTGGGGC Rv: CCAGCAGATGAGCGGGAATAGGACC	62	577
*Agtr1a*	NM_030985.4	Fw: CACCTATGTAAGATCGCTTCGGCC Rv: GGGTATAGCTGGTGAGAATGATAAGG	60	355
*Nos3*	NM_021838.2	Fw: GACCCTCCGCCATCCACAGAGCCTG Rv: GCACCGGGTCTCCTGCCTTGAGTTGG	64	514
*Bdkrb2*	NM_001270713.2	Fw: GCTCATAACGGGACCTTTTCAGAGG Rv: CAGTGGCCTTCTTCTCCGTCTGG	63	700
*Gapdh*	NM_017008.4	Fw: CCCACGGCAAGTTCAACGGC Rv: CCATGTAGGCCATGAGGTCCACC	64	840

NCBI-RS: Reference sequences from National Center for Biotechnology Information of *Rattus norvegicus* genes: angiotensin I converting enzyme (*Ace*), angiotensin II receptor type 1a (*Agtr1a*), nitric oxide synthase 3 (*Nos3*), bradykinin receptor B2 (*Bdkrb2*) and glyceraldehyde-3-phosphate dehydrogenase (*Gapdh*) mRNA. Fw: forward primer, Rv: reverse primer, °C: Celsius grades, bp: base pairs.

## References

[1] James P., Oparil S., Carter B., Cushman W., Dennison-Himmelfarb C., Handler J., Lackland D., LeFevre M., MacKenzie T., Ogedegbe O. (2014). Evidence-based guideline for the management of high blood pressure in adults: Report from the panel members appointed to the Eighth Joint National Committee (JNC 8). JAMA.

[2] Joyner M., Wallin B., Charkoudian N. (2016). Sex differences and blood pressure regulation in humans. Exp. Physiol..

[3] Ferini-Strambi L., Walters A., Sica D. (2014). The relationship among restless legs syndrome (Willis–Ekbom Disease), hypertension, cardiovascular disease, and cerebrovascular disease. J. Neurol..

[4] Tetzner A., Gebolys K., Meinert C., Klein S., Uhlich A., Trebicka J., Villacañas O., Walther T. (2016). G-Protein-coupled receptor MrgD is a receptor for angiotensin-(1-7) involving adenylyl cyclase, cAMP, and phosphokinase A. Hypertension.

[5] Soubrier F., Wei L., Hubert C., Clauser E., Alhenc-Gelas F., Corvol P. (2018). Angiotensin I-converting enzyme (ACE) gene structure and polymorphism: Relation to enzyme function and gene expression. Cellular and Molecular Biology of the Renin-Angiotensin System.

[6] Rajapakse N., Head G., Kaye D. (2016). Say NO to obesity-related hypertension: Role of the l-arginine–nitric oxide pathway. Hypertension.

[7] Li E., Heran B., Wright J. (2014). Angiotensin converting enzyme (ACE) inhibitors versus angiotensin receptor blockers for primary hypertension. Cochrane Database Syst. Rev..

[8] Kessy H., Wang K., Zhao L., Zhou M., Hu Z. (2018). Enrichment and biotransformation of phenolic compounds from litchi pericarps with angiotensin I-converting enzyme (ACE) inhibition activity. LWT Food Sci. Technol..

[9] Knez Hrnčič M., Ivanovski M., Cör D., Knez Ž. (2020). Chia seeds (*Salvia hispanica* L.): An Overview-phytochemical profile, isolation methods, and application. Molecules.

[10] Ullah R., Nadeem M., Khalique A., Imran M., Mehmood S., Javid A., Hussain J. (2016). Nutritional and therapeutic perspectives of Chia (*Salvia hispanica* L.): A review. J. Food Sci. Technol..

[11] Kaur S., Bains K. (2019). Chia (*Salvia hispanica* L.)–a rediscovered ancient grain, from Aztecs to food laboratories. Nutr. Food Sci..

[12] Chan-Zapata I., Arana-Argáez V.E., Torres-Romero J.C., Segura-Campos M.R. (2019). Anti-inflammatory effects of the protein hydrolysate and peptide fractions isolated from *Salvia hispanica* L. seeds. Food Agric. Immunol..

[13] Güzel S., Ülger M., Yusuf Ö.Z.A.Y. (2020). Antimicrobial and antiproliferative activities of Chia (*Salvia hispanica* L.) seeds. Int. J. Second. Metab..

[14] De Falco B., Amato M., Lanzotti V. (2017). Chia seeds products: An overview. Phytochem. Rev..

[15] Marcinek K., Krejpcio Z. (2017). Chia seeds (*Salvia hispanica*): Health promoting properties and therapeutic applications-a review. Rocz. Panstw. Zakł. Hig..

[16] Peiretti P., Gai F. (2009). Fatty acid and nutritive quality of chia (*Salvia hispanica* L.) seeds and plant during growth. Anim. Feed Sci. Technol..

[17] Reyes-Caudillo E., Tecante A., Valdivia-López M. (2008). Dietary fibre content and antioxidant activity of phenolic compounds present in Mexican chia (*Salvia hispanica* L.) seeds. Food Chem..

[18] Ortega A. (2016). Identificación de la actividad inhibitoria presente en extractos de semilla de chía (*Salvia hispanica* L.) sobre la enzima convertidora de angiotensina. Master’s Thesis.

[19] Eriz G., Sanhueza V., Roeckel M., Fernández K. (2011). Inhibition of the angiotensin-converting enzyme by grape seed and skin proanthocyanidins extracted from Vitis vinifera L. cv. País. LWT Food Sci. Technol..

[20] González V. (2011). Efecto hipotensor e inhibición de la actividad de la enzima convertidora de angiotensina I de extractos de semillas de *Salvia hispanica* L. in vitro e in vivo. Master’s Thesis.

[21] Steinkamp-Fenske K., Bollinger L., Völler N., Xu H., Yao Y., Bauer R., Förstermann U., Li H. (2007). Ursolic acid from the chinese herb danshen (*Salvia miltiorrhiza* L.) upregulates eNOS and downregulates Nox4 expression in human endothelial cells. Atherosclerosis.

[22] Segura-Campos M.R., Chel-Guerrero L.A., Rosado-Rubio J.G., Betancur-Ancona D.A. (2016). Biofunctionality of Chia (*Salvia hispanica* L.) Protein Hydrolysates. Functional Properties of Traditional Foods.

[23] Pellegrini M., Lucas-Gonzalez R., Sayas-Barberá E., Fernández-López J., Pérez-Álvarez J., Viuda-Martos M. (2016). Bioaccessibility of Phenolic Compounds and Antioxidant Capacity of Chia (*Salvia hispanica* L.) Seeds. Plant. Foods Hum. Nutr..

[24] Poulios E., Giaginis C., Vasios G.K. (2020). Current State of the Art on the Antioxidant Activity of Sage (Salvia spp.) and Its Bioactive Components. Planta Med..

[25] Zimmermann B.F., Walchc S.G., Tinzoh L.N., Stühlinger W., Lachenmeier D.W. (2011). Rapid UHPLC determination of polyphenols in aqueous infusions of Salvia officinalis L. (sage tea). J. Chromatogr. B. Biomed. Appl..

[26] Jiménez-Ferrer E., Hernández Badillo F., González-Cortazar M., Tortoriello J., Herrera-Ruiz M. (2010). Antihypertensive activity of Salvia elegans Vahl. (Lamiaceae): ACE inhibition and angiotensin II antagonism. J. Ethnopharmacol..

[27] Mostafa M., Gardouh A., Abogresha N., Gad S. (2020). Factorial design, formulation, in vitro and in vivo evaluation of rapid orally disintegrating tablets prepared by sublimation technique using captopril as a model drug. J. Drug. Deliv. Sci. Technol..

[28] Luna-Vital D., González E., Mendoza S., Loarca-Piña G. (2012). Peptides present in the non-digestible fraction of common beans (Phaseolus vulgaris L.) inhibit the angiotensin-I converting enzyme by interacting with its catalytic cavity independent of their antioxidant capacity. Food Funct..

[29] Puga A., López-Oliva S., Trives C., Partearroyo T., Varela-Moreiras G. (2019). Effects of Drugs and Excipients on Hydration Status. Nutrients.

[30] Arenas-Carvajal R., Pachón-Gómez E., Méndez-Callejas G., Guzmán-Avendaño A. (2009). Estudio del efecto inhibitorio de extractos de Salvia scutellarioides sobre la actividad de la enzima convertidora de angiotensina. Univ. Sci..

[31] Chen J., Ryu B., Zhang Y., Liang P., Li C., Zhou C., Yang P., Hong P., Qian Z. (2019). Comparison of an angiotensin-I-converting enzyme inhibitory peptide from tilapia (*Oreochromis niloticus*) with captopril: Inhibition kinetics, in vivo effect, simulated gastrointestinal digestion and a molecular docking study. J. Sci. Food. Agric..

[32] Zhou Z., Wang S., Liu Y., Miao A. (2006). Cryptotanshinone inhibits endothelin-1 expression and stimulates nitric oxide production in human vascular endothelial cells. Biochim. Biophys. Acta.

[33] Kobayashi N., Hara K., Watanabe S., Higashi T., Matsuoka H. (2000). Effect of imidapril on myocardial remodeling in L-NAME-induced hypertensive rats is associated with gene expression of NOS and ACE mRNA. Am. J. Hypertens..

[34] De Gennaro V., Rigamonti A., Fioretti S., Bonomo S., Manfredi B., Ferrario P., Bianchi M., Berti F., Muller E., Rossoni G. (2005). Angiotensin-converting enzyme inhibition and angiotensin AT1-receptor antagonism equally improve endothelial vasodilator function in L-NAME-induced hypertensive rats. Eur. J. Pharmacol..

[35] Schmitt C., Dirsch V. (2009). Modulation of endothelial nitric oxide by plant-derived products. Nitric Oxide.

[36] Wallerath T., Li H., Gödtel-Ambrust U., Schwarz P., Förstermann U. (2005). A blend of polyphenolic compounds explains the stimulatory effect of red wine on human endothelial NO synthase. Nitric Oxide.

[37] Carey R., Siragy H. (2003). Newly recognized components of the renin-angiotensin system: Potential roles in cardiovascular and renal regulation. Endocr. Rev..

[38] Hara K., Kobayashi N., Watanabe S., Tsubokou Y., Matsuoka H. (2001). Effects of quinapril on expression of eNOS, ACE, and AT1 receptor in deoxycorticosterone acetate-salt hypertensive rats. Am. J. Hypertens..

[39] Marketou M., Kontaraki J., Zacharis E., Parthenakis F., Maragkoudakis S., Gavras I., Gavras H., Vardas P. (2014). Differential gene expression of bradykinin receptors 1 and 2 in peripheral monocytes from patients with essential hypertension. J. Hum. Hypertens..

[40] Fox A., Wotherspoon G., McNair K., Hudson L., Patel S., Gentry C., Winter J. (2003). Regulation and function of spinal and peripheral neuronal B1 bradykinin receptor in inflammatory mechanical hyperalgesia. Pain.

[41] Medeiros R., Cabrini D., Ferreira J., Fernandes E., Mori M., Pesquero B., Bader M., Avellar M., Campos M., Calixto B. (2004). Bradykinin B1 receptor expression induced by tissue damage in the rat portal vein a critical role for mitogen-activated protein kinase and nuclear. Circ. Res..

[42] López A., Ortega A. (2014). Caracterización de semilla de chía (*Salvia hispanica* L.): Cuantificación parcial de compuestos fenólicos y capacidad de inhibición de la enzima convertidora de angiotensina I in vitro. (Tesis de licenciatura) Master’s Thesis.

[43] Turck D., Castenmiller J., De Henauw S., Hirsch-Ernst K., Kearney J., Maciuk A., Mangelsdorf I., McArdle H., Naska A., Pelaez C. (2019). Safety of chia seeds (*Salvia hispanica* L.) as a novel food for extended uses pursuant to Regulation (EU) 2015/2283. EFSA J..

[44] Wu J., Aluko R., Muir A. (2002). Improved method for direct high-performance liquid chromatography assay of angiotensin-converting enzyme-catalyzed reactions. J. Chromatogr. A.

[45] Mali V., Mohan V., Bodhankar S. (2012). Antihypertensive and cardioprotective effects of the Lagenaria siceraria fruit in NG-nitro-L-arginine methyl ester (L-NAME) induced hypertensive rats. Pharm. Biol..

[46] Miguel M., Contreras M., Recio I., Aleixandre A. (2009). ACE-inhibitory and antihypertensive properties of a bovine casein hydrolysate. Food Chem..

[47] Singh M., Singh A.K., Pandey P., Chandra S., Singh K.A., Gambhir I.S. (2016). Molecular genetics of essential hypertension. Clin. Exp. Hypertens..

[48] Botzer A., Grossman E., Moult J., Unger R. (2018). A system view and analysis of essential hypertension. J. Hypertens..

[49] Wang L., Cheng F., Hu J., Wang H., Tan N., Li S., Wang X. (2018). Pathway-based gene-gene interaction network modelling to predict potential biomarkers of essential hypertension. BioSystems.

